# The Relationship between *BCMO1* Gene Variants and Macular Pigment Optical Density in Persons with and without Age-Related Macular Degeneration

**DOI:** 10.1371/journal.pone.0089069

**Published:** 2014-02-19

**Authors:** Beatrix Feigl, C. Phillip Morris, Joanne Voisey, Anthony Kwan, Andrew J. Zele

**Affiliations:** 1 Institute of Health and Biomedical Innovation, Queensland University of Technology, Brisbane, QLD, Australia; 2 School of Biomedical Sciences, Queensland University of Technology, Brisbane, QLD, Australia; 3 School of Optometry and Vision Sciences, Queensland University of Technology, Brisbane, QLD, Australia; 4 Queensland Eye Institute, Brisbane, QLD, Australia; 5 Faculty of Health Sciences, University of Queensland, Brisbane, QLD, Australia; Duke University, United States of America

## Abstract

**Background:**

Recent evidence indicates that gene variants related to carotenoid metabolism play a role in the uptake of macular pigments lutein (L) and zeaxanthin (Z). Moreover, these pigments are proposed to reduce the risk for advanced age-related macular degeneration (AMD). This study provides the initial examination of the relationship between the gene variants related to carotenoid metabolism, macular pigment optical density (MPOD) and their combined expression in healthy humans and patients with AMD.

**Participants and Methods:**

Forty-four participants were enrolled from a general population and a private practice including 20 healthy participants and 24 patients with advanced (neovascular) AMD. Participants were genotyped for the three single nucleotide polymorphisms (SNPs) upstream from *BCMO1,* rs11645428, rs6420424 and rs6564851 that have been shown to either up or down regulate beta-carotene conversion efficiency in the plasma. MPOD was determined by heterochromatic flicker photometry.

**Results:**

Healthy participants with the rs11645428 GG genotype, rs6420424 AA genotype and rs6564851 GG genotype all had on average significantly lower MPOD compared to those with the other genotypes (*p*<0.01 for all three comparisons). When combining *BCMO1* genotypes reported to have “high” (rs11645428 AA/rs6420424 GG/rs6564851 TT) and “low” (rs11645428 GG/rs6420424 AA/rs6564851 GG) beta-carotene conversion efficiency, we demonstrate clear differences in MPOD values (p<0.01). In patients with AMD there were no significant differences in MPOD for any of the three *BCMO1* gene variants.

**Conclusion:**

In healthy participants MPOD levels can be related to high and low beta-carotene conversion *BCMO1* genotypes. Such relationships were not found in patients with advanced neovascular AMD, indicative of additional processes influencing carotenoid uptake, possibly related to other AMD susceptibility genes. Our findings indicate that specific *BCMO1* SNPs should be determined when assessing the effects of carotenoid supplementation on macular pigment and that their expression may be influenced by retinal disease.

## Introduction

Macular pigment carotenoids lutein (L), zeaxanthin (Z) and meso-zeaxanthin (MZ) have numerous proposed roles within the eye and brain. These include protection from photochemical damage and oxidative stress [1] and improving visual and cognitive function [2], [3], [4], [5], [6], [7], [8], [9]. A standard clinical protocol for measuring macular pigment optical density (MPOD) using heterochromatic flicker photometry (HFP) has been developed [10]. Dietary and supplementary L and Z intake correlates with serum levels [11], [12], [13] and macular pigment optical density (MPOD) in humans [14], [15], [16], [17], [18]. While factors such as gender [19], age [20], [21], [22], iris colour [23], smoking habits [21] and hereditability [24], [25] have been proposed to modulate macular pigment, genetic variants coding for proteins related to carotenoid metabolism have been linked with macular pigment deposition [26], [27], [28].

It is believed that macular pigments protect from the development of advanced age-related macular degeneration (AMD) with studies showing that AMD patients have lower MPOD compared to healthy participants [29], [30], [31], [32]. These findings gave rise to numerous studies that aimed to determine the effect of L and Z supplementation on AMD progression with varying results [8], [15], [33], [34], [35], [36], [37]. One of the largest studies was the Age-Related Eye Disease Study (AREDS2) [34] that demonstrated no beneficial or harmful effect of L and Z supplementation. However, participants in the quintile with the lowest dietary intake of macular carotenoids had less progression to advanced AMD compared to those who had higher dietary intake when supplementing with the AREDS formula (Vitamin A, E, C beta-carotene, copper and zinc) and L and Z. A potential unknown of the study design was identified as being the effect of simultaneous administration of beta-carotene and L and Z, hence potential competitive absorption of carotenoids [38]. Kostic et al. [38] demonstrated that when lutein and beta-carotene are ingested together, serum peak concentration of lutein is lower in the presence of beta-carotene. Borel et al. [27] further proposed that the enzyme beta-carotene monooxygenase (*BCMO1*) that regulates the conversion from beta-carotene to Vitamin A, may also modulate macular pigment deposition. The conversion activity of *BCMO1* is genetically determined and there is evidence that genotype variants upstream from the *BCMO1* gene affect conversion efficiency [39]. Lietz et al. [39] highlight the importance of genotyping exonic, intronic and intergenic *BCMO1* SNPs to investigate beta-carotene conversion efficiency. In particular, genotypes for three *BCMO1* SNPs (rs11645428, rs6420424 and rs6564851) have been identified, with decreased (rs11645428 GG, rs6420424 AA, rs6564851 GG) and increased (rs11645428 AA, rs6420424 GG, rs6564851 TT) beta-carotene conversion efficiency but the relationship with macular pigment levels was not evaluated in that study [39]. A large observational study demonstrated a strong association between MPOD and the *BCMO1* SNP (rs11645428) in a healthy cohort of postmenopausal women [26]. Another recent study confirmed these findings but found the strongest association between MPOD and the *BCMO1* SNP rs6564851 [28]. However, this study did not show an association between *BCMO1* variants and macular pigment levels after L and Z supplementation. Neither study investigated the combined effect of all three *BCMO1* SNPs (rs11645428, rs6420424 and rs6564851) that may act synergistically to alter the catalytic activity of *BCMO1* and consequently alter pigment deposition in the macula. Moreover, these studies were performed in healthy participants and *BCMO1* effects may not only vary in different populations [39] but also with retinal disease.

The aim of this study was therefore to determine the relationship between MPOD and three SNPs (rs11645428 and rs6420424, rs6564851) upstream from the *BCMO1* gene. Our hypothesis is that participants with “high” and “low” β-carotene conversion genotypes will have high and low MPOD levels, respectively. We investigated a group of healthy participants and patients with advanced neovascular macular degeneration. The reason for including the latter group was to determine whether *BCMO1* gene variants are also associated with disease progression. Our hypothesis is that given participants have already developed advanced AMD, the *BCMO1* SNPs will impact less on MPOD as other environmental and genetic factors may dominate.

## Methods

### Ethics Statement

The Queensland University of Technology (QUT) Human Research Ethics Committee approved this study. The study was conducted in accordance with the guidelines of the QUT Human Research Ethics Committee and the tenets of the Declaration of Helsinki. Written informed consent was obtained from each participant.

### Participants

In total, 44 participants were recruited from a general population (healthy participants with no eye disease) and a private practice (patients with age-related macular degeneration) and tested for macular pigment optical density (MPOD). All participants were of European ancestry except for two AMD patients who were of Asian ancestry. Participants were genotyped for the SNPs rs11645428, rs6420424 and rs6564851 upstream of the *BCMO1* gene. Of the 44 patients, 20 participants were assigned to the healthy group (mean age 56±5 yrs, 8 female and 12 male). The healthy participants had best corrected visual acuities of greater than 1.0, normal intraocular pressure (<21 mmHg), no signs of anterior segment or retinal or optic nerve disease and normal central retinal thickness within reported normative values as assessed with optical coherence tomography (OCT, Stratus, Zeiss, Germany). Twenty-four participants were assigned to the patient group with neovascular AMD (mean age 80±7 yrs, 14 female and 10 male). All AMD patients had steady fixation and most of them had a visual acuity of greater than 0.3 (20/50) as suggested by the MPOD manufacturer. Those participants who had a visual acuity less than 0.3 (n = 3) were still able to fixate and perform the MPOD successfully as has been previously demonstrated in patients with reduced visual acuity in AMD [29], [40]. All the AMD patients were classified as AREDS level 4b [41] and were undergoing intravitreal injection with anti-vascular endothelial growth factor (anti-VEGF) treatment according to established procedures [42]. None of the participants were taking supplements containing either lutein or zeaxanthin at the time of the study, and none were current smokers.

### Macular Pigment Optical Density (MPOD) Determination

Macular Pigment Optical Density and ophthalmic routine assessment (visual acuity, indirect ophthalmoscopy, intraocular pressure and OCT) were performed by the same investigators (ophthalmologists BF, AK and optometry staff who were blind to the genotypes of each participant). Macular pigment optical density was determined by heterochromatic flicker photometry (Macular Metrics II, LLC, Providence, USA) with a 0.5° stimulus presented in the fovea and a 2° stimulus presented at 7° in the paracentral area [43]. The stimulus consisted of a flickering light that alternates between a 460 nm light that is maximally absorbed by macular pigment, and a 564 nm light that is not absorbed by macular pigment. All participants performed four measurements at both retinal locations. The flicker frequency was adjusted individually before starting the test. The amount of 460 nm light required to achieve minimum flicker (iso-luminance between the blue and green stimuli) is used to calculate a participant’s MPOD (in density units, D.U.) which is the log ratio of the amount of blue light absorbed centrally to that absorbed peripherally. Patients in the AMD group performed another four measurements at both retinal locations at a second visit (within 3 months of treatment).

### Genotyping

The DNA was extracted from saliva samples (Oragene DNA self-collection kit, DNA Genotek Inc, Ottawa, Ontario, Canada) according to established procedures used previously in our laboratories [44], [45]. DNA was then genotyped for the SNPs rs11645428, rs6420424 and rs6564851 commercially at the Australian Genome Research Facility (AGRF) using the Sequenom MassARRAY genotyping system.

### Statistical Analysis

Data were analysed with a statistical package (IBM SPSS Statistics 21). Comparisons between MPOD levels and genotypes were made using one-way ANOVA and post-hoc analysis.

## Results

Initial data screening indicated the data were normally distributed. Forty-three out of 44 participants were successfully genotyped for rs11645428 and rs6564851 and all 44 participants were genotyped for rs6420424. Genotypes were in Hardy-Weinberg equilibrium (HWE) as computed using Utility Programs for Analysis of Genetic Linkage [46].

Table 1 shows genotype and allele frequencies for the three *BCMO1* SNPs and mean MPOD. Alleles were within reported frequencies from other studies [26], [39]. For example, this study found a minor allele frequency (MAF) of 35% for rs11645428 and Meyer’s et al. [26] found a MAF of 33%. Lietz et al. [39] summarised allele frequencies of rs6420424 and rs6564851 from different ethnic groups including their own cohort of 28 female volunteers. They found a MAF of 45% for rs6420424 compared to 48% in our study and 48% for rs6564851 compared to 47% in our study.

**Table 1:** Genotype and allele frequencies of the *BCMO1* SNPs rs11645428, rs6420424 and rs6564851 and mean MPOD.

		Genotype		
Group	SNP and MPOD	GG	GA	AA
Healthy	*BCMO1* rs11645428	45%(n = 9)	35%(n = 7)	20%(n = 4)
	Mean MPOD D.U. ± SD	0.38±0.2	0.51±0.1	0.71±0.2
	*BCMO1* rs6420424	25%(n = 6)	50%(n = 12)	25%(n = 6)
	Mean MPOD D.U. ± SD	0.67±0.2	0.48±0.1	0.33±0.2
		**GG**	**GT**	**TT**
	*BCMO1* rs6564851	25%(n = 5)	50%(n = 10)	25%(n = 5)
	Mean MPOD D.U. ± SD	0.29±0.2	0.48±0.1	0.72±0.2
		**GG**	**GA**	**AA**
AMD	*BCMO1* rs11645428	43%(n = 10)	48%(n = 11)	9%(n = 2)
	Mean MPOD D.U. ± SD	0.47±0.2	0.45±0.1	0.39±0.1
	*BCMO1* rs6420424	29%(n = 7)	50%(n = 12)	21%(n = 5)
	Mean MPOD D.U. ± SD	0.4±0.1	0.46±0.1	0.52±0.2
		**GG**	**GT**	**TT**
	*BCMO1* rs6564851	17%(n = 4)	53%(n = 12)	30%(n = 7)
	Mean MPOD D.U. ± SD	0.48±0.2	0.46±0.1	0.38±0.1
		**Alleles**		
		**G**	**A**	**–**
Healthyand AMD	*BCMO1* rs11645428	65%(n = 56)	35%(n = 30)	–
	*BCMO1* rs6420424	52%(n = 46)	48%(n = 42)	–
		**G**	**T**	**–**
	*BCMO1* rs6564851	47%(n = 40)	53%(n = 46)	–

**Figure 1:**
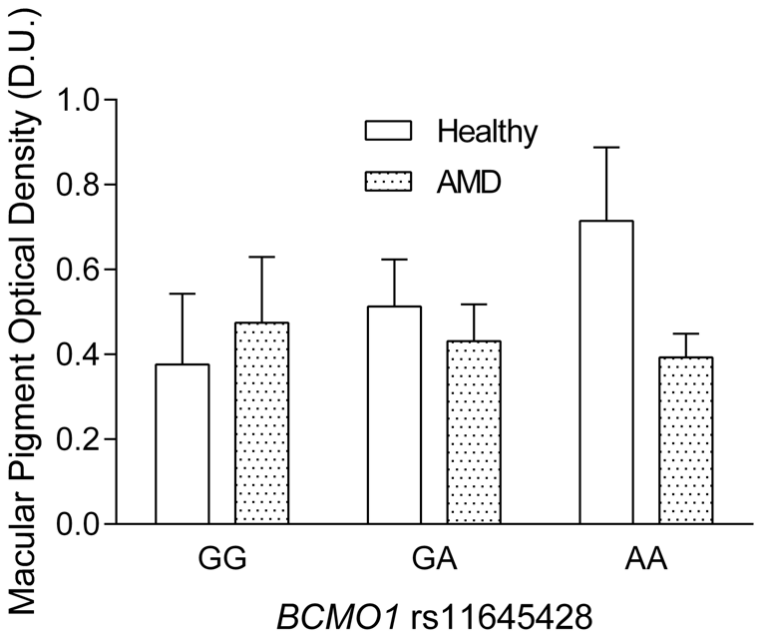
MPOD as a function of *BCMO1* rs11645428 (error bars indicate+SD). Healthy participants with the GG genotype show on average significantly lower MPOD compared to those with the AA genotype. There were no significant differences between genotypes in the AMD group.

**Figure 2:**
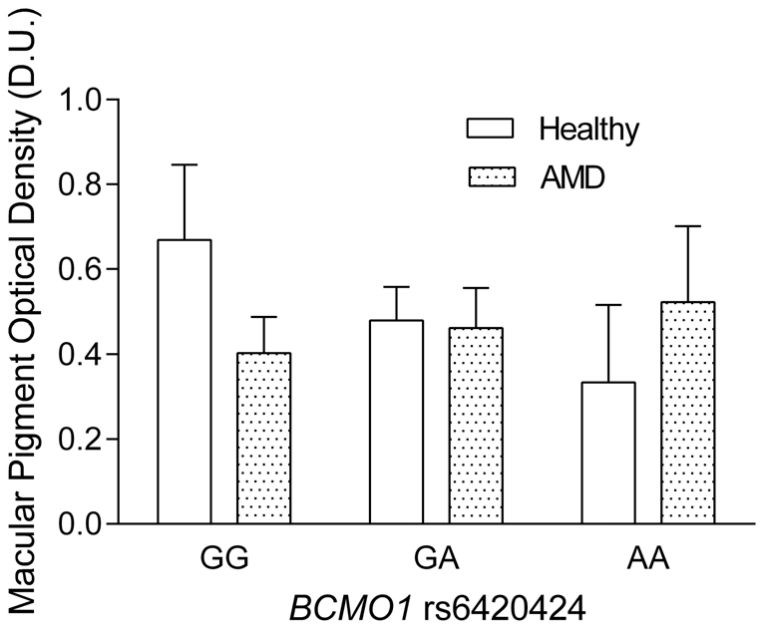
MPOD as a function of *BCMO1* rs6420424 (error bars indicate+SD). Healthy participants with the GG genotype show on average significantly higher MPOD compared to those with the AA genotype. There were no significant differences between genotypes in the AMD group.

**Figure 3:**
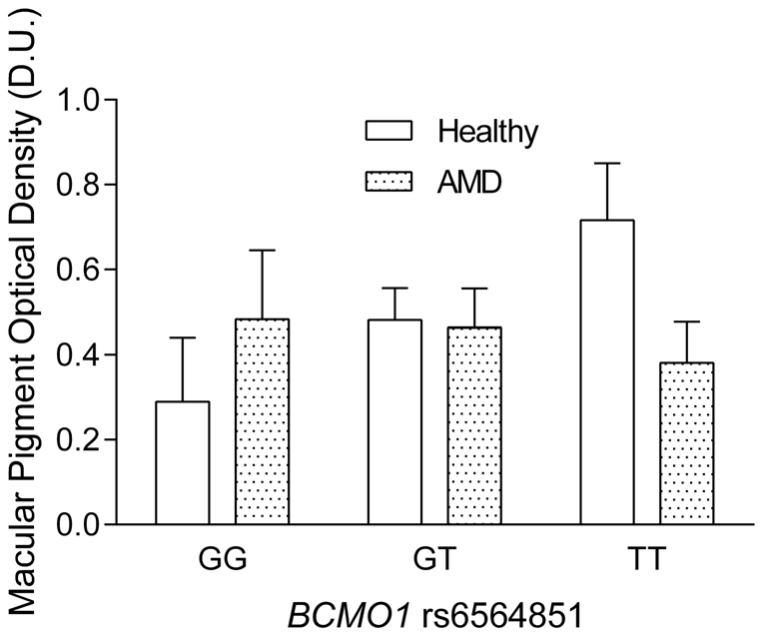
MPOD as a function of *BCMO1* rs6564851 (error bars indicate+SD). Healthy participants with the TT genotype show on average significantly higher MPOD compared to those with the GG genotype. There were no significant differences between genotypes in the AMD group.

In the healthy group, there was a significant difference between rs11645428 genotypes (F_2,19_ = 7.1, *p*<0.01) with the mean MPOD values being on average lower for those with the GG genotype (0.38±0.2 D.U.) compared to those with the GA (0.51±0.1 D.U.) and the AA genotypes (0.71±0.2 D.U.) (Figure 1). Post-hoc analysis demonstrated that the rs11645428 GG genotype was significantly lower compared to the AA genotype (*p* = 0.002) and that the GA genotype was significantly lower than the AA genotype (*p* = 0.05). There was also a significant difference between the rs6420424 genotypes (F_2,19_ = 7.8, *p*<0.01) where those with the GG genotype had higher MPOD levels compared to those with the AA genotype. The average MPOD for the GG, GA and AA genotypes were 0.67±0.2 D.U., 0.48±0.1 D.U. and 0.33±0.2 D.U., respectively. Post-hoc analysis demonstrated that participants with the GG genotype had significantly higher MPOD levels compared to GA (*p* = 0.03) and AA (p<0.01) (Figure 2). Analysis of rs6564851 in healthy participants demonstrated significant differences in mean MPOD (F_2,19_ = 15.5, *p*<0.001), with the TT genotype showing significantly higher MPOD (0.72±0.2 D.U.), compared to the GT (0.48±0.1 D.U.) and GG (0.29±0.2 D.U.) genotypes. Post-hoc analysis revealed that the TT genotype was significantly higher than the GT and GG genotype (p<0.01) (Figure 3).

In the patients with age-related macular degeneration there was on average no significant difference in MPOD between the two visits within the three months of anti-VEGF treatment (visit one: mean MPOD = 0.47±0.1 D.U. and visit 2: mean MPOD = 0.44±0.2 D.U., *p* = 0.4). The average MPOD of these two time points was taken for further analysis. There was no significant difference in MPOD between the rs11645428 genotypes (F_2,22_ = 0.4, *p* = 0.7), the rs6420424 genotypes (F_2,23_ = 1.6, *p* = 0.2) or the rs6564851 genotypes (F_2,22_ = 1.0, *p* = 0.3) in the AMD patients. The average MPOD in the rs11645428 and rs6420424 GG, GA and AA genotypes were 0.47±0.2 D.U., 0.45±0.1 D.U., 0.39±0.1 D.U. and 0.4±0.1 D.U., 0.46±0.1 D.U., 0.52±0.2 D.U., respectively. The average MPOD for the rs6564851 TT, GT and GG genotypes was 0.38±0.1 D.U., 0.46±0.1 D.U and 0.48±0.2 D.U, respectively (Figure 1–3) (Table 1).

**Figure 4:**
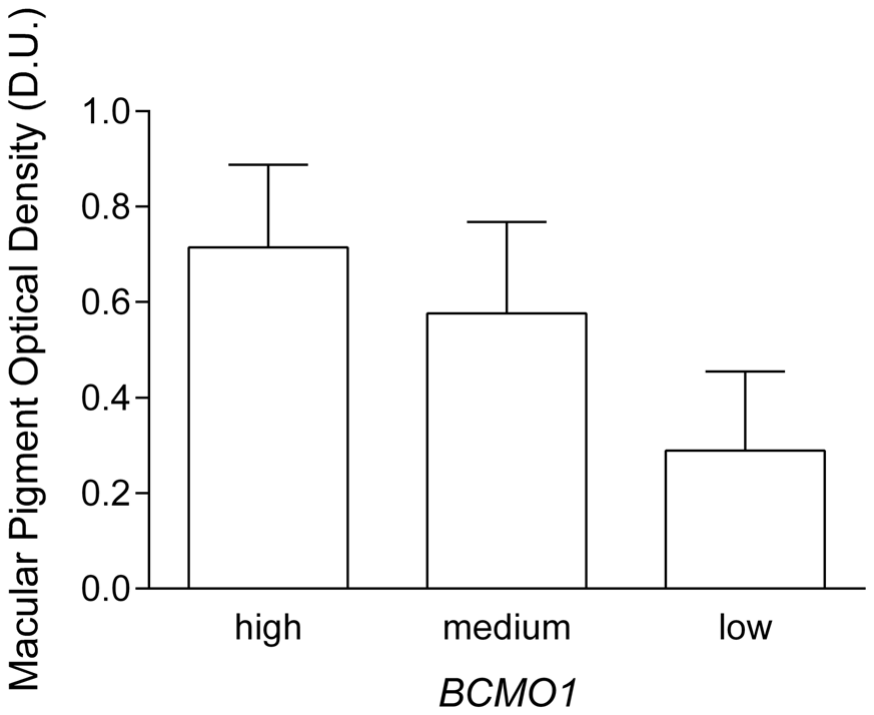
MPOD as a function of “high conversion” (high) (rs11645428 AA/rs6420424 GG/rs6564851 TT), “low conversion” (low) (rs11645425 GG/rs6420424 AA/rs6564851 GG) and “medium conversion” (medium) (rs11645425/rs6420424/rs6564851 remaining genotypes) triple genotypes (error bars indicate+SD). Healthy participants with the “high conversion” genotype had significantly higher MPOD compared with the “low conversion” genotype.

We determined rs11645428, rs6420424 and rs6564851 “triple genotypes” based on the previously reported BCMO1 beta-carotene conversion efficiency, that is, those who should have high- or low-conversion efficiency based on their *BCMO1* genotypes [39]. Interestingly, the healthy participants MPOD values reflected plasma conversion efficiency, with the GG/AA/GG (rs11645428/rs6420424/rs6564851) low conversion triple genotypes having low MPOD values, those with the AA/GG/TT high conversion triple genotype had significantly higher MPOD values while the remaining genotypes had intermediate MPOD values (Figure 4). There was a significant difference between the three high, low and intermediate MPOD groups (F_2,16_ = 6.8, *p* = 0.01). Data show that healthy persons with the AA/GG/TT genotypes have on average significantly higher MPOD levels (0.72±0.2 D.U.) compared to those with the GG/AA/GG genotypes (MPOD = 0.29±0.2 D.U.) (*p*<0.01). Those healthy participants with the remaining genotypes showed significantly higher MPOD (0.58±0.2 D.U.) compared to the GG/AA/GG genotype with the lowest MPOD (*p* = 0.02) (Figure 4) (Table 2).

**Table 2:** Genotype frequencies of *BCMO1* triple genotypes (rs11645428, rs6420424 and rs6564851) and mean MPOD.

Triplegenotypes	AA/GG/TT	GG/AA/GG	Remaining genotypes
Frequency	24% (n = 4)	29% (n = 5)	47% (n = 8)
Mean MPODD.U. ± SD	0.72±0.2	0.29±0.2	0.58±0.2

## Discussion

The data demonstrate that *BCMO1* SNPs and their predicted effects on beta-carotene conversion efficiency in the plasma [39] can be related to MPOD. We show that healthy participants fall into high, low and intermediate MPOD values based on combined *BCMO1* rs11645428/rs6420424/rs6564851 genotypes. We demonstrate that healthy participants with rs11645428 GG, rs6420424 AA and rs6564851 GG “low conversion” genotypes had also low MPOD levels and that those with rs11645428 AA, rs6420424 GG and rs6564851 TT “high conversion” genotypes had high MPOD levels. All the remaining homozygous and heterozygous genotypes had MPOD levels that were between the “high” and “low” conversion genotype groups. Hence the findings support our hypothesis that the increased *BCMO1* β-carotene conversion efficiency in the plasma is reflected in increased MPOD and that decreased conversion results in decreased MPOD.

The *BCMO1* gene encodes an enzyme that cleaves β-carotenoids to produce vitamin A. It has been hypothesised that its reduced activity due to variants in the *BCMO1* gene has an effect on retinal levels of L and Z and that *BCMO1* may modulate concentration of lutein in the macula [27]. The mechanism by which a change in enzymatic activity affects carotenoid uptake however, is not clear. During et al. [47] suggested an antagonising effect by showing differential absorption patterns of carotenoids in a cellular *in vitro* model. Our findings suggest that decreased conversion of β-carotene and hence increased β-carotene availability may cause competition with lutein uptake, thereby decreasing MPOD. Conversely, increased β-carotene conversion and reduced availability may improve uptake of macular pigments in healthy humans.

In the cohort with age-related macular degeneration, MPOD did not differ between genotypes or triple genotypes. This is consistent with our hypothesis and suggests that other factors may be involved. In particular, in advanced AMD where there is a change in retinal anatomy, retinal carotenoid binding proteins [48], [49], [50] may be altered, hence affecting MPOD. Additional environment and genetic factors are influential in the evolution of AMD and while macular pigments may protect from environmental risk, this function may be “overridden” by a strong genetic predisposition for AMD. All our patients had advanced neovascular AMD and macular pigment was not a protective factor in this cohort. Complement factor H (*CFH*) genotypes have been identified as major contributors to the risk of developing AMD [51], [52] and we found that the AMD patients with the homozygous *CFH* high risk genotype had on average lower MPOD (0.43±0.1 D.U.) compared to the low risk genotypes (0.47±0.1 D.U) (data not shown, *p* = 0.2). This is in support of another study that demonstrated reduced macular pigment in a small cohort of healthy participants who were homozygous for *CFH* risk genotypes [53]. Our findings are further supported by a recent large study in women that investigated the relationship of *BCMO1* genotypes with macular pigment optical density in a sample of patients who predominantly had early AMD [54]. While Meyers et al. [54] did not investigate combined *BCMO1* genotypes as done in this study, they demonstrated a relationship between MPOD and one of the *BCMO1* genotypes we investigated. The strongest relationship however, was found between MPOD and other genes related to carotenoid status (*ABCA1*, *ALDH3A2* and *BCO2*) [54]. Meyers et al. [54] acknowledge that carotenoid pathway genes need to be evaluated in relation to early and advanced AMD risk in prospective studies. Our study therefore provides evidence of a differing impact of *BCMO1* genotypes on MPOD based on AMD stage.

Another finding was that repeated anti-VEGF treatment did not affect MPOD levels over a short period of three months. Anti-VEGF therapy can cause toxic photoreceptor damage [55], and macular pigment can be found in outer photoreceptor segments. The effect of anti-VEGF on MPOD however, is unknown and studies have only considered the effects of laser treatment on MPOD [29]. Our findings may have importance for recovery of vision in patients undergoing anti-VEGF treatment given the role of macular pigment in visual performance with studies demonstrating that macular pigments enhance contrast sensitivity, chromatic discrimination and mesopic visual function [2], [5], [6], [56]. However, it remains to be shown whether supplementation of macular pigments is useful in advanced AMD for improvement of visual function rather than protection, in particular when considering that the retinal uptake of pigments may be affected.

Our study reflects emerging evidence of the significant effect of gene variants modifying MPOD levels and that this effect may be different in patients with retinal disease. Our study confirms large association studies of *BCMO1* gene variants with MPOD in healthy participants [26] [28]. It is known that these SNPs are associated with high or low serum beta-carotene levels [39] but none of these studies investigated the combined effects of these SNPs on MPOD. Also, these effects were not evaluated in different cohorts with and without AMD. Additionally, our study suggests that competitive uptake of carotenoids requires further assessment in different population cohorts with and without retinal disease.

## References

[1] Khachik F, Bernstein PS, Garland DL (1997) Identification of lutein and zeaxanthin oxidation products in human and monkey retinas. Invest Ophthalmol Vis Sci 38: 1802–1811. 9286269

[2] Loughman J, Akkali MC, Beatty S, Scanlon G, Davison PA, et al (2010) The relationship between macular pigment and visual performance. Vision Res 50: 1249–1256. 20394766 10.1016/j.visres.2010.04.009

[3] Hammond BR Jr, Fletcher LM, Elliott JG (2013) Glare disability, photostress recovery, and chromatic contrast: relation to macular pigment and serum lutein and zeaxanthin. Invest Ophthalmol Vis Sci 54: 476–481. 23211814 10.1167/iovs.12-10411

[4] Kijlstra A, Tian Y, Kelly ER, Berendschot TT (2012) Lutein: more than just a filter for blue light. Prog Ret Eye Res 31: 303–315. 10.1016/j.preteyeres.2012.03.002 22465791

[5] Kvansakul J, Rodriguez-Carmona M, Edgar DF, Barker FM, Kopcke W, et al (2006) Supplementation with the carotenoids lutein or zeaxanthin improves human visual performance. Ophthal Physiol Opt 26: 362–371. 10.1111/j.1475-1313.2006.00387.x 16792735

[6] Rodriguez-Carmona M, Kvansakul J, Harlow JA, Kopcke W, Schalch W, et al (2006) The effects of supplementation with lutein and/or zeaxanthin on human macular pigment density and colour vision. Ophthal Physiol Opt 26: 137–147. 10.1111/j.1475-1313.2006.00386.x 16460314

[7] Murray IJ, Makridaki M, van der Veen RL, Carden D, Parry NR, et al (2013) Lutein supplementation over a one year period in early AMD might have a mild beneficial effect on visual acuity; the CLEAR study. Invest Ophthalmol Vis Sci 54: 1781–1788. 23385792 10.1167/iovs.12-10715

[8] Ma L, Yan SF, Huang YM, Lu XR, Qian F, et al (2012) Effect of lutein and zeaxanthin on macular pigment and visual function in patients with early age-related macular degeneration. Ophthalmology 119: 2290–2297. 22858124 10.1016/j.ophtha.2012.06.014

[9] Feeney J, Finucane C, Savva GM, Cronin H, Beatty S, et al (2013) Low macular pigment optical density is associated with lower cognitive performance in a large, population-based sample of older adults. Neurobiol Aging 34: 2449–2456. 23769396 10.1016/j.neurobiolaging.2013.05.007

[10] Snodderly DM, Mares JA, Wooten BR, Oxton L, Gruber M, et al (2004) Macular pigment measurement by heterochromatic flicker photometry in older subjects: the carotenoids and age-related eye disease study. Invest Ophthalmol Vis Sci 45: 531–538. 14744895 10.1167/iovs.03-0762

[11] Hammond BR Jr, Curran-Celentano J, Judd S, Fuld K, Krinsky NI, et al (1996) Sex differences in macular pigment optical density: relation to plasma carotenoid concentrations and dietary patterns. Vision Res 36: 2001–2012. 8759440 10.1016/0042-6989(95)00290-1

[12] Carroll YL, Corridan BM, Morrissey PA (1999) Carotenoids in young and elderly healthy humans: dietary intakes, biochemical status and diet-plasma relationships. Eur J Clin Nutr 53: 644–653. 10477252 10.1038/sj.ejcn.1600827

[13] Rosenthal JM, Kim J, de Monasterio F, Thompson DJ, Bone RA, et al (2006) Dose-ranging study of lutein supplementation in persons aged 60 years or older. Invest Ophthalmol Vis Sci 47: 5227–5233. 17122107 10.1167/iovs.05-1513

[14] Renzi LM, Hammond BR, Dengler M, Roberts R (2012) The relation between serum lipids and lutein and zeaxanthin in the serum and retina: results from cross-sectional, case-control and case study designs. Lipids Health Dis 11: 33. 22375926 10.1186/1476-511X-11-33 PMC3310786

[15] Koh HH, Murray IJ, Nolan D, Carden D, Feather J, et al (2004) Plasma and macular responses to lutein supplement in subjects with and without age-related maculopathy: a pilot study. Exp Eye Res 79: 21–27. 15183097 10.1016/j.exer.2004.03.001

[16] Thurnham DI (2007) Macular zeaxanthins and lutein – a review of dietary sources and bioavailability and some relationships with macular pigment optical density and age-related macular disease. Nutr Res Rev 20: 163–179. 19079868 10.1017/S0954422407842235

[17] Connolly EE, Beatty S, Thurnham DI, Loughman J, Howard AN, et al (2010) Augmentation of macular pigment following supplementation with all three macular carotenoids: an exploratory study. Curr Eye Res 35: 335–351. 20373901 10.3109/02713680903521951

[18] Mares JA, LaRowe TL, Snodderly DM, Moeller SM, Gruber MJ, et al (2006) Predictors of optical density of lutein and zeaxanthin in retinas of older women in the Carotenoids in Age-Related Eye Disease Study, an ancillary study of the Women's Health Initiative. Am J Clin Nutr 84: 1107–1122. 17093164 10.1093/ajcn/84.5.1107

[19] Hammond BR Jr, Caruso-Avery M (2000) Macular pigment optical density in a Southwestern sample. Invest Ophthalmol Vis Sci 41: 1492–1497. 10798668

[20] Nolan JM, Kenny R, O'Regan C, Cronin H, Loughman J, et al (2010) Macular pigment optical density in an ageing Irish population: The Irish Longitudinal Study on Ageing. Ophthalmic Res 44: 131–139. 20516725 10.1159/000315531

[21] Dietzel M, Zeimer M, Heimes B, Claes B, Pauleikhoff D, et al (2011) Determinants of macular pigment optical density and its relation to age-related maculopathy: results from the Muenster Aging and Retina Study (MARS). Invest Ophthalmol Vis Sci 52: 3452–3457. 21296816 10.1167/iovs.10-6713

[22] Berendschot TT, van Norren D (2005) On the age dependency of the macular pigment optical density. Exp Eye Res 81: 602–609. 16024015 10.1016/j.exer.2005.03.019

[23] Howells O, Eperjesi F, Bartlett H (2013) Macular Pigment Optical Density in Young Adults of South Asian Origin. Invest Opthalmol Vis Sci 54: 2211–2219. 10.1167/iovs.12-10957 23471890

[24] Liew SH, Gilbert CE, Spector TD, Mellerio J, Marshall J, et al (2005) Heritability of macular pigment: a twin study. Invest Ophthalmol Vis Sci 46: 4430–4436. 16303930 10.1167/iovs.05-0519

[25] Hammond CJ, Liew SH, Van Kuijk FJ, Beatty S, Nolan JM, et al (2012) The heritability of macular response to supplemental lutein and zeaxanthin: a classic twin study. Invest Ophthalmol Vis Sci 53: 4963–4968. 22700713 10.1167/iovs.12-9618 PMC3410678

[26] Meyers KJ, Johnson EJ, Bernstein PS, Iyengar SK, Engelman CD, et al (2013) Genetic determinants of macular pigments in women of the Carotenoids in Age-Related Eye Disease Study. Invest Ophthalmol Vis Sci 54: 2333–2345. 23404124 10.1167/iovs.12-10867 PMC3626525

[27] Borel P (2012) Genetic variations involved in interindividual variability in carotenoid status. Mol Nutr Food Res 56: 228–240. 21957063 10.1002/mnfr.201100322

[28] Yonova-Doing E, Hysi PG, Venturini C, Williams KM, Nag A, et al (2013) Candidate gene study of macular response to supplemental lutein and zeaxanthin. Exp Eye Res 115: 172–177. 23891863 10.1016/j.exer.2013.07.020 PMC3819993

[29] Obana A, Hiramitsu T, Gohto Y, Ohira A, Mizuno S, et al (2008) Macular carotenoid levels of normal subjects and age-related maculopathy patients in a Japanese population. Ophthalmology 115: 147–157. 18166409 10.1016/j.ophtha.2007.02.028

[30] Bernstein PS, Zhao DY, Wintch SW, Ermakov IV, McClane RW, et al (2002) Resonance Raman measurement of macular carotenoids in normal subjects and in age-related macular degeneration patients. Ophthalmology 109: 1780–1787. 12359594 10.1016/s0161-6420(02)01173-9 PMC3079575

[31] Raman R, Biswas S, Gupta A, Kulothungan V, Sharma T (2012) Association of macular pigment optical density with risk factors for wet age-related macular degeneration in the Indian population. Eye 26: 950–957. 22562185 10.1038/eye.2012.69 PMC3396169

[32] Kaya S, Weigert G, Pemp B, Sacu S, Werkmeister RM, et al (2012) Comparison of macular pigment in patients with age-related macular degeneration and healthy control subjects - a study using spectral fundus reflectance. Acta Ophthalmol 90: e399–403. 23035764 10.1111/j.1755-3768.2012.02423.x

[33] Richer S, Stiles W, Statkute L, Pulido J, Frankowski J, et al (2004) Double-masked, placebo-controlled, randomized trial of lutein and antioxidant supplementation in the intervention of atrophic age-related macular degeneration: the Veterans LAST study (Lutein Antioxidant Supplementation Trial). Optometry 75: 216–230. 15117055 10.1016/s1529-1839(04)70049-4

[34] Age-Related Eye Disease Study 2 Research Group (2013) Lutein+zeaxanthin and omega-3 fatty acids for age-related macular degeneration: the Age-Related Eye Disease Study 2 (AREDS2) randomized clinical trial. JAMA 309: 2005–2015. 23644932 10.1001/jama.2013.4997

[35] Dawczynski J, Jentsch S, Schweitzer D, Hammer M, Lang GE, et al (2013) Long term effects of lutein, zeaxanthin and omega-3-LCPUFAs supplementation on optical density of macular pigment in AMD patients: the LUTEGA study. Graefe's Arch Clin Exp Ophthalmol 251: 2711–2723. 23695657 10.1007/s00417-013-2376-6

[36] Trieschmann M, Beatty S, Nolan JM, Hense HW, Heimes B, et al (2007) Changes in macular pigment optical density and serum concentrations of its constituent carotenoids following supplemental lutein and zeaxanthin: the LUNA study. Exp Eye Res 84: 718–728. 17306793 10.1016/j.exer.2006.12.010

[37] Weigert G, Kaya S, Pemp B, Sacu S, Lasta M, et al (2011) Effects of lutein supplementation on macular pigment optical density and visual acuity in patients with age-related macular degeneration. Invest Ophthalmol Vis Sci 52: 8174–8178. 21873668 10.1167/iovs.11-7522

[38] Kostic D, White WS, Olson JA (1995) Intestinal absorption, serum clearance, and interactions between lutein and beta-carotene when administered to human adults in separate or combined oral doses. Am J Clin Nutr 62: 604–610. 7661123 10.1093/ajcn/62.3.604

[39] Lietz G, Oxley A, Leung W, Hesketh J (2012) Single nucleotide polymorphisms upstream from the beta-carotene 15,15'-monoxygenase gene influence provitamin A conversion efficiency in female volunteers. J Nutr 142: 161S–165S. 22113863 10.3945/jn.111.140756

[40] Stringham JM, Hammond BR, Nolan JM, Wooten BR, Mammen A, et al (2008) The utility of using customized heterochromatic flicker photometry (cHFP) to measure macular pigment in patients with age-related macular degeneration. Exp Eye Res 87: 445–453. 18778703 10.1016/j.exer.2008.08.005

[41] Age-Related Eye Disease Study Research Group (2001) The age-related eye disease study system for classifying age-related macular degeneration from stereoscopic color fundus photographs: the Age-Related Eye Disease Study Report Number 6. Am J Ophthalmol 132: 668–681. 11704028 10.1016/s0002-9394(01)01218-1

[42] Mitchell P, Korobelnik JF, Lanzetta P, Holz FG, Prunte C, et al (2010) Ranibizumab (Lucentis) in neovascular age-related macular degeneration: evidence from clinical trials. Br J Ophthalmol 94: 2–13. 19443462 10.1136/bjo.2009.159160

[43] Wooten BR, Hammond BR Jr, Land RI, Snodderly DM (1999) A practical method for measuring macular pigment optical density. Invest Ophthalmol Vis Sci 40: 2481–2489. 10509640

[44] Feigl B, Cao D, Morris CP, Zele AJ (2011) Persons with age-related maculopathy risk genotypes and clinically normal eyes have reduced mesopic vision. Invest Ophthalmol Vis Sci 52: 1145–1150. 20881291 10.1167/iovs.10-5967 PMC3053098

[45] Feigl B, Morris CP, Brown B, Zele AJ (2012) Relationship Among CFH and ARMS2 Genotypes, Macular Pigment Optical Density, and Neuroretinal Function in Persons Without Age-Related Macular Degeneration. Arch Ophthalmol 130: 1402–1409. 22777494 10.1001/archophthalmol.2012.1940

[46] Ott J (1988) Utility programs for analysis of genetic linkage, Program, HWE version 1.10. New York: Columbia University.

[47] During A, Hussain MM, Morel DW, Harrison EH (2002) Carotenoid uptake and secretion by CaCo-2 cells: beta-carotene isomer selectivity and carotenoid interactions. J Lipid Res 43: 1086–1095. 12091493 10.1194/jlr.m200068-jlr200

[48] Vachali P, Besch BM, Gonzalez-Fernandez F, Bernstein PS (2013) Carotenoids as Possible Interphotoreceptor Retinoid-binding Protein (IRBP) Ligands: A Surface Plasmon Resonance (SPR) Based Study. Arch Biochem Biophys 539: 181–186. 23876239 10.1016/j.abb.2013.07.008 PMC3818380

[49] Li B, Vachali P, Frederick JM, Bernstein PS (2011) Identification of StARD3 as a lutein-binding protein in the macula of the primate retina. Biochemistry 50: 2541–2549. 21322544 10.1021/bi101906y PMC3070171

[50] Bhosale P, Larson AJ, Frederick JM, Southwick K, Thulin CD, et al (2004) Identification and characterization of a Pi isoform of glutathione S-transferase (GSTP1) as a zeaxanthin-binding protein in the macula of the human eye. J Biol Chem 279: 49447–49454. 15355982 10.1074/jbc.M405334200

[51] Seddon JM, Francis PJ, George S, Schultz DW, Rosner B, et al (2007) Association of CFH Y402H and LOC387715 A69S with progression of age-related macular degeneration. JAMA 297: 1793–1800. 17456821 10.1001/jama.297.16.1793

[52] Schaumberg DA, Hankinson SE, Guo Q, Rimm E, Hunter DJ (2007) A prospective study of 2 major age-related macular degeneration susceptibility alleles and interactions with modifiable risk factors. Arch Ophthalmol 125: 55–62. 17210852 10.1001/archopht.125.1.55

[53] Loane E, Nolan JM, McKay GJ, Beatty S (2011) The association between macular pigment optical density and CFH, ARMS2, C2/BF, and C3 genotype. Exp Eye Res 93: 592–598. 21816153 10.1016/j.exer.2011.07.005

[54] Meyers KJ, Mares JA, Igo RP Jr, Truitt B, Liu Z, et al.. (2013) Genetic Evidence for Role of Carotenoids in Age-Related Macular Degeneration in the Carotenoids in Age-Related Eye Disease Study (CAREDS). Invest Ophthalmol Vis Sci doi: 10.1167/iovs.13–13216. [Epub ahead of print]. 10.1167/iovs.13-13216 PMC3908680 24346170

[55] Quaggin SE (2012) Turning a blind eye to anti-VEGF toxicities. J Clin Invest 122: 3849–3851. 23093785 10.1172/JCI65509 PMC3498935

[56] Nolan JM, Loughman J, Akkali MC, Stack J, Scanlon G, et al (2011) The impact of macular pigment augmentation on visual performance in normal subjects: COMPASS. Vision Res 51: 459–469. 21237188 10.1016/j.visres.2010.12.016

